# Bufalin inhibits gastric cancer invasion and metastasis by down-regulating Wnt/ASCL2 expression

**DOI:** 10.18632/oncotarget.24157

**Published:** 2018-01-11

**Authors:** Jie Wang, Han Cai, Yue Xia, Shiying Wang, Likai Xing, Chao Chen, Yong Zhang, Jie Xu, Peihao Yin, Yiming Jiang, Ronghua Zhao, Qingshong Zuo, Teng Chen

**Affiliations:** ^1^ Department of General Surgery, Putuo Hospital, Shanghai University of Traditional Chinese Medicine, Shanghai 200062, China; ^2^ Department of Gastroenterology, Longhua Hospital, Shanghai University of Traditional Chinese Medicine, Shanghai 200032, China; ^3^ Department of Medical, Virogin Biotech Ltd., Vancouver, British Columbia V6S 2L9, Canada; ^4^ Shanghai Putuo Central School of Clinical Medicine, Anhui Medical University, Shanghai 200062, China

**Keywords:** bufalin, gastric cancer, invasion and metastasis, Wnt/ASCL2 signaling, EMT

## Abstract

Achaete-scute-like 2 (ASCL2) is a transcription factor containing a basic helix-loop-helix (bHLH) domain and is a downstream target of Wnt signaling in intestinal stem cells. Bufalin is the primary active ingredient in Chan Su, a traditional Chinese medicine obtained from the skin and parotid venom glands of toads. The purpose of this study was to research the anti-invasion and anti-metastasis activity of bufalin in gastric cancer and to identify the potential mechanism. Bufalin inhibited gastric cancer cell invasion and metastasis, suppressed cancer cell colony formation, and inhibited the growth of subcutaneous xenografted tumors in nude mice. Furthermore, bufalin inhibited ASCL2 expression and down-regulated the expression of invasion-related genes such as MMP2, MMP9, and vimentin, thereby suppressing epithelial-mesenchymal transition (EMT) in gastric cancer. A Wnt signaling inhibitor (XAV939) down-regulated invasion and the expression of ASCL2, β-catenin, and vimentin but up-regulated E-cadherin expression. In nude mice, bufalin inhibited the tumorigenic behavior of gastric cancer cells, induced cancer cell apoptosis, and regulated invasion-related gene expression. Together, our results suggest that bufalin arrests invasion and metastasis and that its mechanism of action may involve down-regulating Wnt/ASCL2 expression.

## INTRODUCTION

Gastric cancer (GC) is one of the most common malignancies worldwide [1]. In China, GC ranks second-highest in incidence and mortality [2]. Surgery is still the first choice for the treatment of GC. However, most patients are diagnosed at an advanced stage, and the available treatments are usually ineffective; more than half of radically resected GC patients experience local relapse or distant metastasis, and the majority receive a diagnosis of GC when the tumor has already disseminated. Thus, the median survival rarely exceeds 12 months, and the 5-year survival is less than 10% [3]. Therefore, it is of great significance to investigate the mechanism of GC progression and metastasis and to identify potent drugs or therapeutic targets.

Bufalin is a bioactive polyhydroxysteroid isolated from Venenum Bufonis, also called Chan Su, a traditional Chinese medicine obtained from the skin and parotid venom glands of toads [4]. Recent experimental studies have indicated that Chan Su and its active compound bufalin have significant antitumor activity in various cancers. For example, bufalin can inhibit cancer cell proliferation [5, 6], induce cancer cell apoptosis and autophagy [7, 8], inhibit cancer metastasis and invasion [9, 10], and reverse multidrug resistance [11]. Studies had found that bufalin inhibits GC proliferation, induces apoptosis, and reverses cisplatin resistance [12, 13]. However, the anti-invasion and anti-metastasis activity of bufalin in GC is still not clear.

Achaete-scute-like 2 (ASCL2), a transcription factor containing a basic helix-loop-helix (bHLH) domain, is a member of the achaete-scute complex-like (ASCL) gene family, which is also referred to as the ‘achaete-scute complex homolog’ family, ‘achaete-scute family basic helix-loop-helix transcription factor’, or mammalian achaete-scute homologs (MASH); there are five family members (ASCL1-ASCL5) [14, 15]. ASCL2 is a master regulator of intestinal stem cell identity and a Wnt target gene. ASCL2 also forms an autoactivating loop that results in an on/off expression pattern; this loop translates a Wnt gradient into a discrete transcriptional decision. In this manner, ASCL2 forms a transcriptional switch that is both Wnt responsive and Wnt dependent to define stem cell identity [16, 17]. Studies have reported that abnormal ASCL2 expression is involved in cell proliferation, invasion, migration, and epithelial-mesenchymal transition (EMT) in colorectal cancer (CRC) [18, 19]. In addition, elevated ASCL2 expression is associated with the metastasis of osteosarcoma and lung squamous cell carcinoma and predicts poor prognosis in these patients [20, 21].

Data have also indicated that ASCL2 expression is up-regulated and markedly hypomethylated in GC tissues compared with adjacent normal tissues. ASCL2 expression levels were inversely correlated with GC patient survival. Ectopic overexpression of ASCL2 can increase cell growth and promote resistance to 5-fluorouracil in GC cells [22]. In our preliminary study, we screened a GC cell line with high ASCL2 expression and found that bufalin down-regulated ASCL2 expression. However, it remained unknown whether down-regulating ASCL2 expression affects GC invasion and metastasis and whether bufalin inhibits GC invasion and metastasis by regulating ASCL2 expression. Therefore, in this study, we down-regulated ASCL2 expression with lentiviral-based RNAi, observed the invasion and migration of transfected GC cells, and studied the effect of bufalin on GC both *in vitro* and *in vivo*.

## RESULTS

### Bufalin inhibited gastric cancer proliferation and colony formation

**Figure 1:**
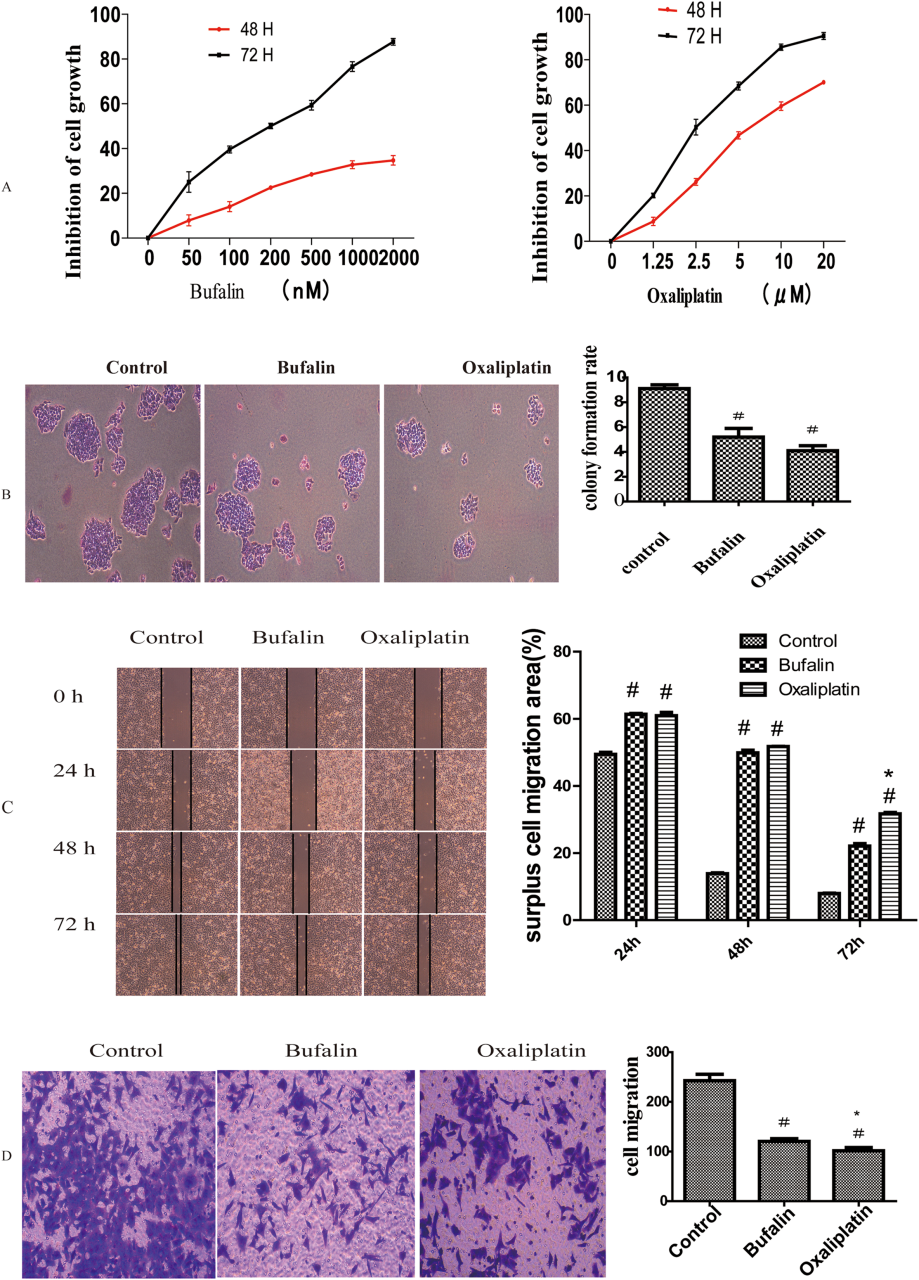
Effect of bufalin on GC cell proliferation and invasion **(A)** MTT assays showing the inhibitory effect of bufalin and oxaliplatin on cell proliferation. **(B)** Colony formation assays after treatment with bufalin, oxaliplatin, or normal saline (control). **(C)** Effect of bufalin and oxaliplatin on cell invasion in wound healing assays. **(D)** Effect of bufalin on cell invasion in transwell assays. All data are representative of at least three independent experiments. ^#^*P*<0.01 compared with the control group, ^*^P<0.05 compared with the bufalin group.

To study the effect of bufalin on GC, we used the most frequently used chemotherapeutic, oxaliplatin, as a positive control. MTT assay results showed that after treatment with different concentrations of bufalin (0, 50, 100, 200, 400, 1000, or 2000 nM) or oxaliplatin (0, 1.25, 2.5, 5, 10, or 20 μM), the proliferation of GC cells (AGS) was obviously restrained, and cell growth was inhibited in a dose- and time-dependent manner. The half-inhibitory concentration (IC50) of bufalin at 48 h in AGS cells was approximately 72 nM, while that for oxaliplatin was 8.56 μM (Figure 1A). Therefore, we used these concentrations in our subsequent study. The colony formation experiment showed that bufalin and oxaliplatin inhibited GC colony clone, but there was no difference between bufalin and oxaliplatin (Figure 1B).

### Bufalin suppressed the invasion and metastasis ability of GC cells

We used wound healing assays to analyze the impact of bufalin on GC cell migration. Migration was obviously suppressed after treatment with bufalin or oxaliplatin in 24h, 48h and 72h compared with control group. But there was no difference between the bufalin and oxaliplatin group in 24h, 48h, while in the time of 72h the Oxaliplatin surpress function was more obviously than bufalin (Figure 1C). To further ascertain the effect of bufalin on tumor cell invasion and metastasis, we performed transwell experiments and found that cell invasion decreased after treatment with bufalin or oxaliplatin; however, oxaliplatin had a stronger effect (Figure 1D).

### ASCL2 promoted gastric cancer cell invasion and metastasis

**Figure 2:**
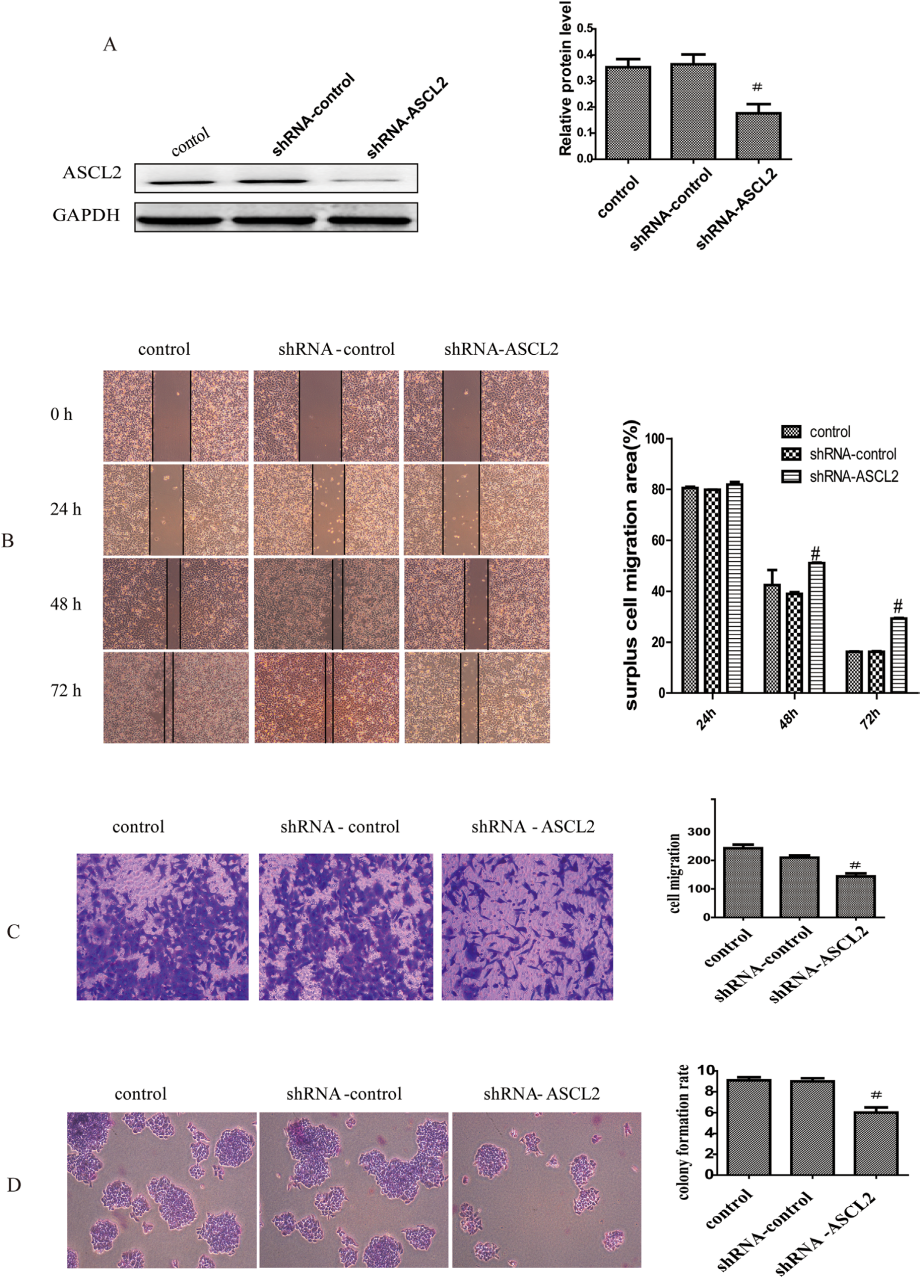
ASCL2 promotes gastric cancer cell invasion and metastasis **(A)** ASCL2 expression after transfection of ASCL2-shRNA. **(B-C)** Effect of down-regulating ASCL2 on cell invasion in wound healing and transwell assays. **(D)** Effect of ASCL2 down-regulation on colony formation ability. All data are representative of at least three independent experiments. ^#^*P*<0.05 compared with the control group.

To study the function of ASCL2 in GC invasion and metastasis, a lentiviral vector containing RNAi targeting the coding region of human ASCL2 mRNA (ASCL2-shRNA) was constructed. The vector was transfected into AGS cells, and ASCL2 expression was detected by western blotting. ASCL2 expression was obviously reduced in shRNA-ASCL2-transfected AGS cells (Figure 2A), and AGS cells with ASCL2 down-regulated were successfully constructed. The migration and invasion of the transfected AGS cells were evaluated using wound healing assays and transwell assays, respectively. Compared with the control vector, the ASCL2-shRNA vector obviously decreased the migration (Figure 2B) and invasion (Figure 2C) of AGS cells. The colony formation assay showed that down-regulation of ASCL2 expression markedly restrained AGS colony formation (Figure 2D).

### Bufalin down-regulated ASCL2 expression

**Figure 3:**
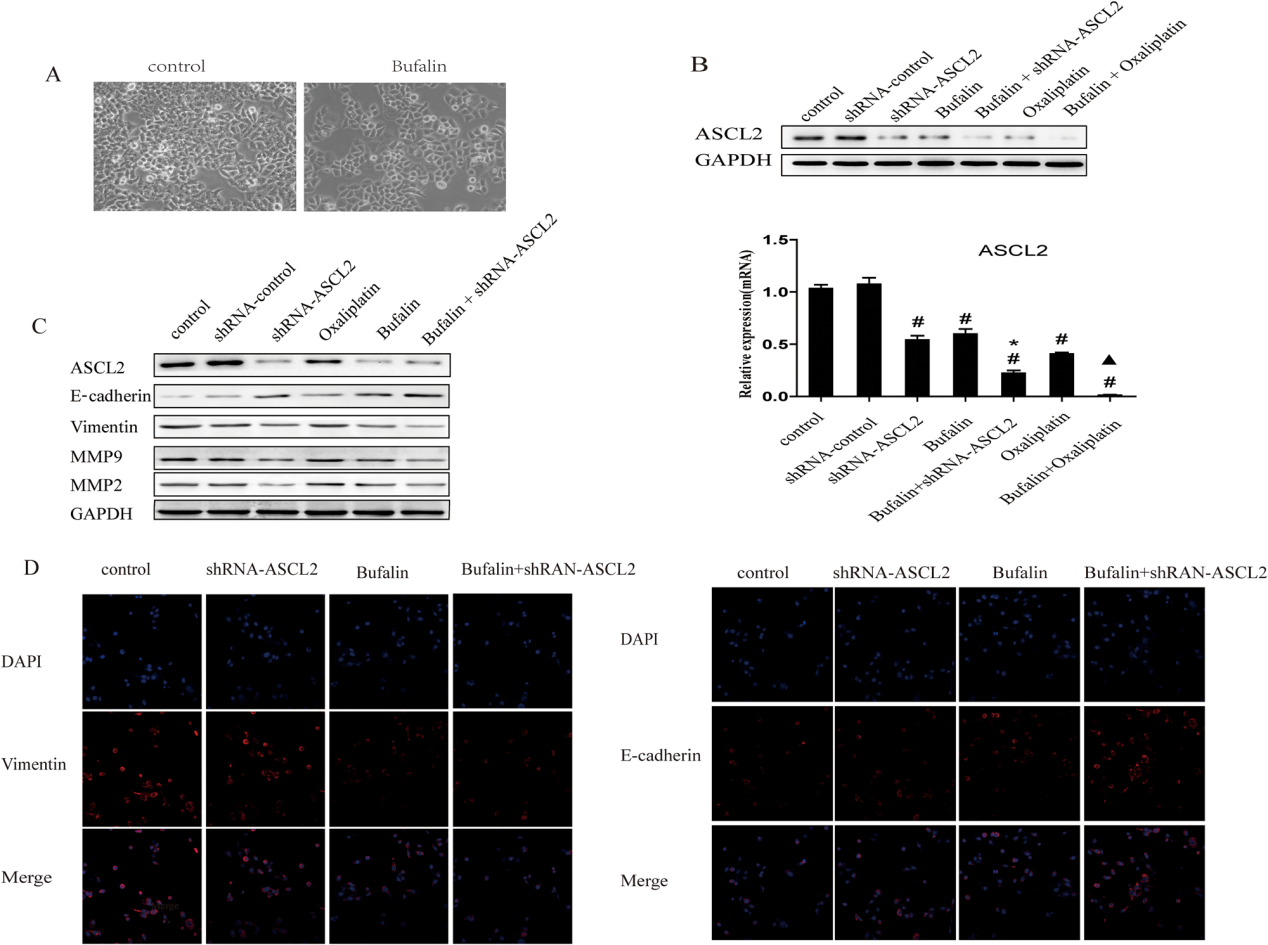
Effect of bufalin on ASCL2 and EMT. **(A)** Morphological changes after bufalin treatment. **(B)** Bufalin down-regulated ASCL2 expression both in protein and mRNA. **(C)** Bufalin affected invasion and metastasis by suppressing EMT. **(D)** Immunofluorescent detection of vimentin and E-cadherin expression, Nucleus were counterstained using DAPI.

The above studies indicated that bufalin inhibits GC invasion and migration and that down-regulating ASCL2 expression has the same consequences. Therefore, the question arose as to whether there is any relation between bufalin-mediated anti-cancer activity and ASCL2 expression. Thus, we determined the effect of bufalin on ASCL2. Western blotting and RT-PCR results showed that bufalin treatment down-regulated ASCL2 expression, and this effect was enhanced by co-treatment with shRNA-ASCL2. Furthermore, bufalin and oxaliplatin had the same activity; co-treatment with both compounds further decreased ASCL2 expression (Figure 3B).

### Bufalin inhibited GC cell invasion and metastasis by suppressing EMT

Since RNAi targeting ASCL2 inhibited the invasion and migration of GC cells, we studied the underlying mechanism. Western blot results showed that ASCL2 down-regulation inhibited the expression of MMP2 and MMP9, increased the expression of the epithelial marker E-cadherin and decreased the expression of the mesenchymal marker vimentin (Figure 3C). Western blot assays were performed to determine whether bufalin affects these genes and showed that bufalin treatment had the same outcome as RNAi targeting ASCL2; combined treatment of bufalin and shRNA-ASCL2 led to a further increased in E-cadherin expression and a further decrease in MMP2, MMP9 and vimentin expression (Figure 3C).

Immunofluorescent detection demonstrated decreasing expression of vimentin and an increasing expression of E-cadherin in the shRNA-ASCL2 AGS cells and bufalin treated cells compared with the control group. Combination treatments with bufalin and shRNA-ASCL2 had greater effects than the single agents(Figure 3D). Moreover, morphological changes were evident after bufalin treatment; GC cells lost their fibroblast-like appearance and acquired an epithelial morphology (Figure 3A).

### Bufalin inhibited the Wnt/β-catenin signaling pathway

**Figure 4:**
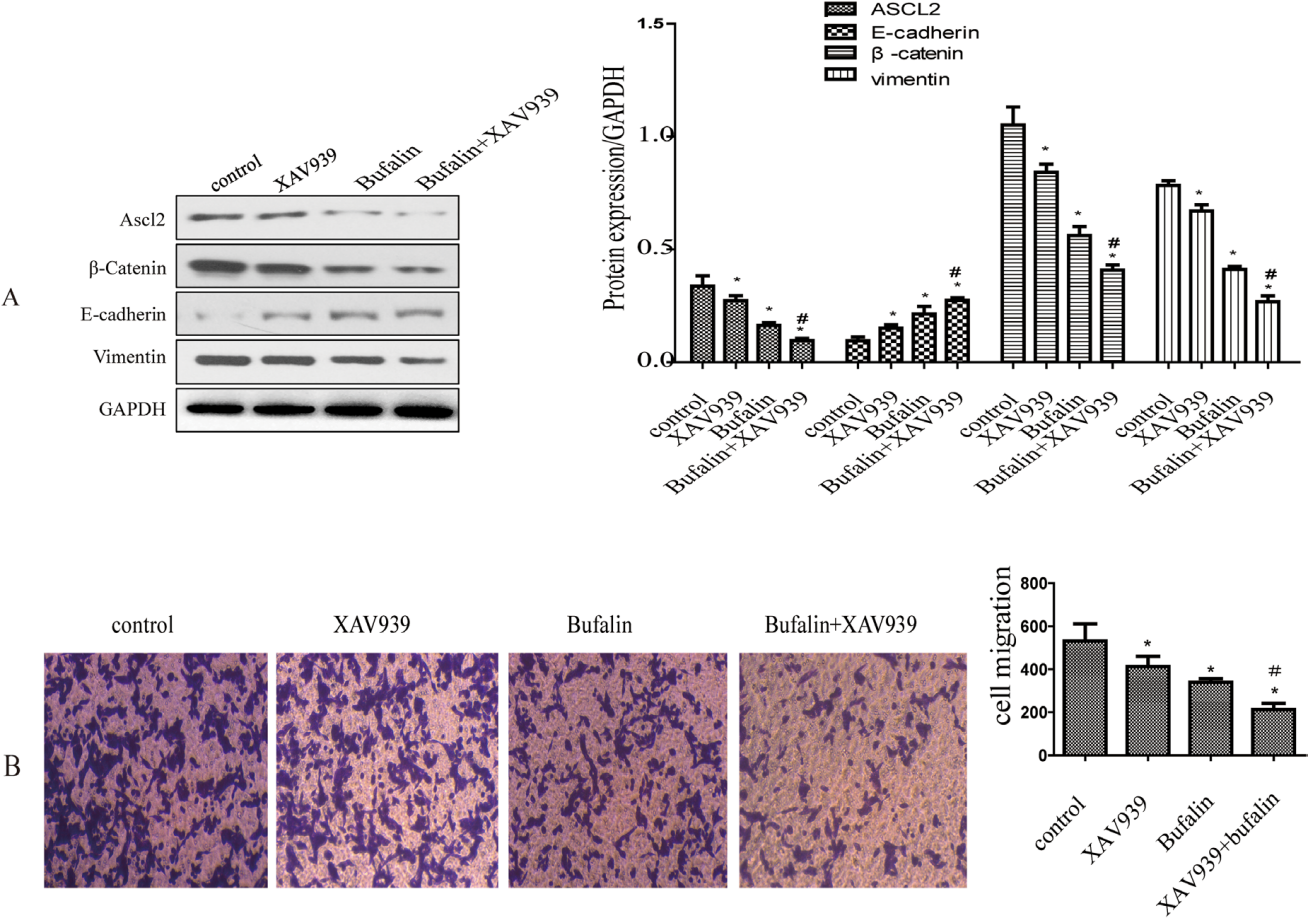
Bufalin inhibits the Wnt/β-catenin signaling pathway. **(A)** The effect of bufalin and XAV939 on ASCL2, β-catenin, E-cadherin and vimentin expression. **(B)** The effect of bufalin and XAV939 on gastric cancer invasion. All data are representative of at least three independent experiments. ^*^P<0.01 compared with the control group, ^#^*P*<0.05 compared with the XAV939 and Bufalin groups.

Studies have shown that ASCL2 is a master regulator of intestinal stem cell identity and a Wnt target gene [16]. Therefore, we inhibited Wnt signaling with XAV939 to determine whether this influences ASCL2 expression. Western blot results showed that after XAV939 treatment, β-catenin and ASCL2 expression were down-regulated, while the expression of the EMT-related gene E-cadherin was up-regulated (Figure 4A). Transwell assays showed that GC cell invasion was inhibited when Wnt signaling was blocked (Figure 4B). Next, we treated GC cells with bufalin and observed that ASCL2, β-catenin and vimentin expression was down-regulated; the down-regulation of these genes was more obvious when bufalin was combined with XAV939 (Figure 4A), and E-cadherin expression was higher in cells treated with the combination than in those treated with bufalin only (Figure 4A). Transwell assays also showed stronger inhibition of cell invasion by bufalin combined with the Wnt inhibitor than by bufalin alone (Figure 4B).

### The effect of ASCL2 and Bufalin on tumorigenic ability

**Figure 5:**
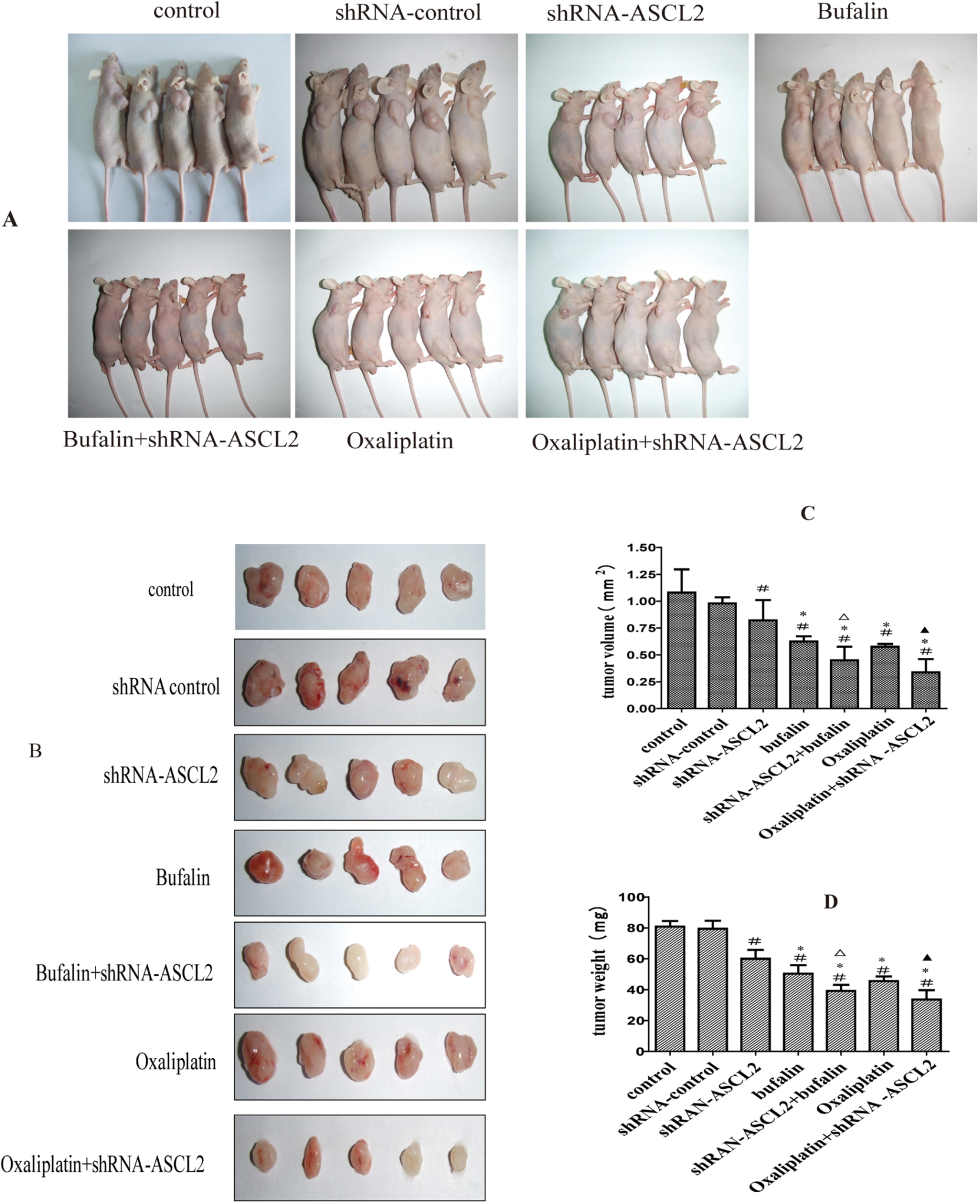
The effect of bufalin on tumorigenic activity **(A)** Subcutaneous transplant model in nude mice. **(B)** Tumors extracted after subcutaneous growth. **(C)** Tumor volume of each group. **(D)** Tumor weight of each group. ^#^P<0.01 compared with the control group, ^*^P<0.05 compared with the shRNA-ASCL2 group, ^Δ^*P*<0.05 compared with the bufalin group, ^▲^*P*<0.01 compared with the oxaliplatin group.

To further investigate ASCL2 function and bufalin activity in GC, we generated a subcutaneous transplant model in nude mice (Figure 5A). First, we observed the effect of ASCL2 on the tumorigenic ability of GC cells; tumorigenesis was markedly inhibited upon down-regulation of ASCL2 expression (Figure 5B). The tumor volume was smaller and the tumor weight was lighter compared with the values in the control group (Figure 5C–5D). Then, we injected bufalin intraperitoneally into animals in the normal cancer cell group and the shRNA-ASCL2 cell group; the control group received normal saline (NS), and the positive control group received oxaliplatin. Bufalin and oxaliplatin inhibited the growth of transplanted tumors compared with control and shRNA-ASCL2, but there was no obvious difference between the bufalin and oxaliplatin groups (Figure 5A–5D). In addition, compared with the groups that received bufalin or oxaliplatin alone, the shRNA-ASCL2 combination treatment groups had smaller and lighter tumors (Figure 5A–5D).

### The effect of bufalin on invasion- and metastasis-related genes *in vivo*

**Figure 6:**
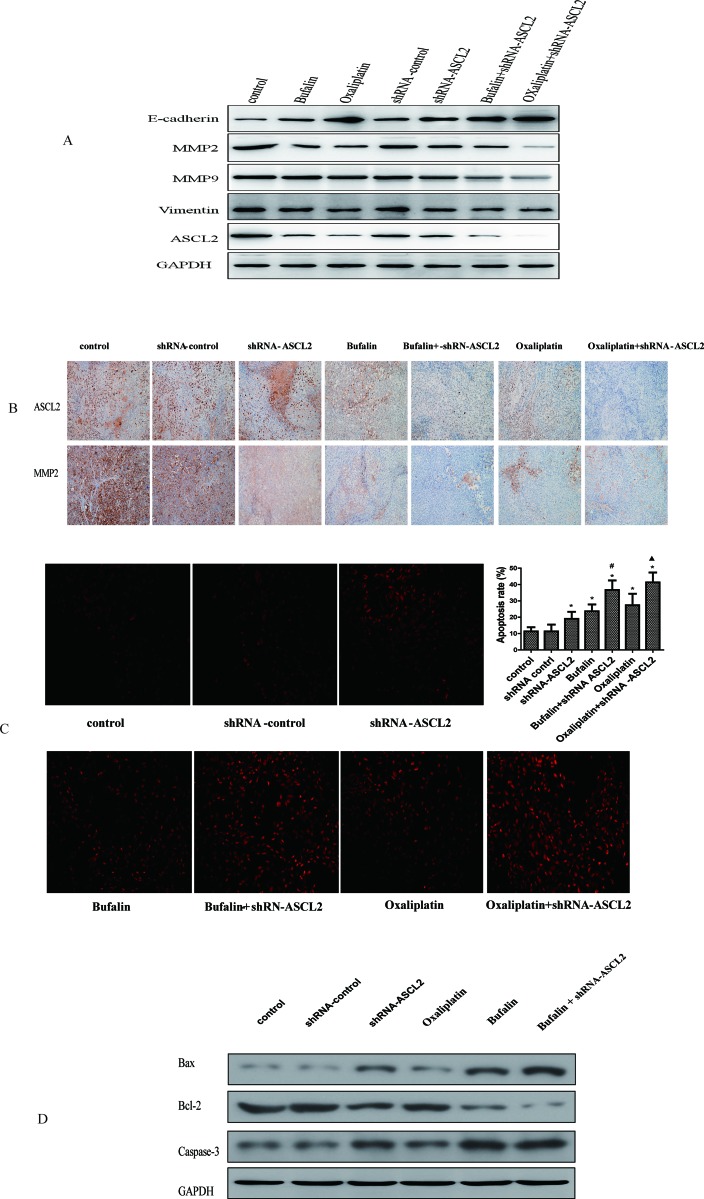
The effect of bufalin on related genes and apoptosis *in vivo* **(A)** Invasion- and metastasis-related genes expression *in vivo* (western-blot). **(B)** ASCL2 and MMP2 exprssion in tumor tissue (IHC). **(C)** The effect of bufalin on GC apoptosis (TUNEL). **(D)** The effect of bufalin on apoptosis relate genes of Bcl-2, Bax and Caspase-3. ^*^P<0.05 compared with the control group, ^#^P<0.01 compared with the bufalin group, ^▲^*P*<0.01 compared with the oxaliplatin group.

Next, we observed the effect of bufalin on ASCL2 and related genes in mouse tumors. Western blot analysis showed that ASCL2, MMP2 and vimentin expression were down-regulated after treatment with bufalin and oxaliplatin (Figure 6A), and these results were verified by immunohistochemistry (IHC) (Figure 6B). Moreover, when bufalin or oxaliplatin was combined with shRNA-ASCL2, MMP2 and vimentin expression was further inhibited, and MMP9 expression was down-regulated; however, there were no difference between bufalin and oxaliplatin (Figure 6A). We also examined E-cadherin expression in the tumors; western blots showed that E-cadherin was up-regulated in the bufalin, oxaliplatin group and shRNA-ASCL2 groups (Figure 6A). Combination treatments with shRNA-ASCL2 had greater effects than the single agents (Figure 6A).

### Bufalin induced gastric cancer apoptosis

Finally, we ascertained the effect of bufalin on GC apoptosis. TUNEL assay results showed that down-regulating ASCL2 expression induced GC apoptosis; the apoptosis rate was higher in the shRNA-ASCL2 group than in the control group. In addition, the apoptosis rate in the bufalin and oxaliplatin groups was higher than that in the control group, while combination treatments with shRNA-ASCL2 led to increased apoptosis compared with bufalin or oxaliplatin alone (Figure 6C). Then we examed the expression of apoptosis relate genes, western-blot result showed that down-regulating ASCL2 expression or treat with bufalin can inhibite the expression of Bcl-2, and up-regulate the expression of Bax and Caspase-3. When combination treatments with shRNA-ASCL2 and bufalin the expression of Bcl-2 almost died out, while the expression of Bax and Caspase-3 were much higher (Figure 6D).

## DISCUSSION

Bufalin is a bioactive polyhydroxysteroid isolated from Venenum Bufonis, also called Chan Su, a well-known traditional Chinese medicine widely used in cancer treatment in China [4, 23]. Recent studies have shown that bufalin has anti-cancer activity in various cancers, such as breast cancer [24], osteosarcoma [5], colon cancer [11, 25], lung cancer [26], pancreatic cancer [27], bladder cancer [28] and hepatocellular carcinoma [9]. Studies had found that bufalin inhibits GC proliferation, induces apoptosis and reverses cisplatin resistance [12, 13]. However, the effects of bufalin on GC invasion and metastasis were not clear. In this study, we found that bufalin inhibits GC proliferation and metastasis. Bufalin treatment decreased colony formation, invasion and migration (Figure 1). In the vivo study, bufalin inhibited the growth of subcutaneous transplanted tumor cells in a nude mouse model (Figure 5). In this study, we used oxaliplatin as a positive control. We observed that bufalin had the same effects as oxaliplatin on GC, but the dose of bufalin was much lower; therefore, bufalin may have potential as a chemotherapeutic inhibitor of GC invasion and metastasis.

Ascl2 is a master regulator of intestinal stem cell identity and a Wnt target gene [16, 17]. Studies have shown that abnormal expression of ASCL2 can enhance the invasion and metastasis of CRC cells *in vitro*; that overexpression of ASCL2 correlates with distant metastasis, tumor size and poor overall survival in CRC patients; and that high expression of ASCL2 promotes EMT in CRC [18, 19, 29]. In addition, elevated ASCL2 expression is associated with the metastasis of osteosarcoma and lung squamous cell carcinoma and predicts poor prognosis of these patients [20, 21]. A previous study found that ASCL2 expression was up-regulated and markedly hypomethylated in GC tissues and that ectopic overexpression of ASCL2 increased cell growth and promoted resistance to 5-fluorouracil in GC cells [22]. In our study, we first determined the effect of ASCL2 on GC invasion and metastasis. Down-regulating ASCL2 expression with lentiviral vector-based RNAi inhibited GC cell colony formation. Wound healing and transwell assays showed that ASCL2 down-regulation led to decreased GC cell invasion and migration (Figure 2). Furthermore, down-regulating ASCL2 decreased the tumorigenicity of GC cells, and tumor volume and weight were both reduced (Figure 5).

Therefore, to investigate whether bufalin-mediated inhibition of GC cell invasion and metastasis is related to ASCL2 expression, we detect ASCL2 protein after treatment with bufalin. ASCL2 was obviously down-regulated after cells were treated with bufalin and oxaliplatin. In addition, when these compounds were combined with shRNA-ASCL2, the expression of ASCL2 was nearly abolished (Figure 3B). Therefore, we speculate that bufalin inhibits GC cell invasion in a manner related to down-regulating ASCL2 expression.

Invasion and metastasis are the primary reasons for the poor prognosis of GC patients [3]. Similar to other cancers, GC cells degrade the basement membrane and extracellular matrix and migrate into adjacent areas before intravasating into blood and/or lymphatic vessels, circulating to the target organ site, extravasating into target organ tissue and proliferating in target organs. These steps are mediated by various factors and processes, including growth factors, proteolytic degradation of extracellular matrix, cell-cell adhesion, cytoskeleton remodeling and changes in gene expression [30]. MMP2 and MMP9 are members of the matrix metalloproteinase (MMP) family of proteolytic enzymes, and a previous study reported that MMPs are considerably elevated in malignant tumors [31]. Expression of these enzymes is a major characteristic of the malignant invasion and metastasis of cancer cells, as these enzymes degrade the ECM and may promote the penetration of cancer cells into the basement membrane [32]. Our study showed that bufalin and ASCL2 knockdown both inhibited the expression of MMP2 and MMP2, with more pronounced results upon combining bufalin and shRNA-ASCL2 (Figure 3C); these results were verified in animal experiments (Figure 6A).

Another reason for metastasis is EMT, one of the key processes in cancer progression and metastasis. Upon undergoing EMT, cancer cells lose cell-cell connections, cell-matrix contact, and normal epithelial polarity while gaining mesenchymal characteristics to enable migration and invasion into the surrounding matrix [33–35]. During EMT, the expression of epithelial cell markers such as E-cadherin is down-regulated, while mesenchymal markers such as vimentin and N-cadherin are up-regulated. Vimentin, a member of the type III intermediate filament (IF) protein family, plays an important role in maintaining cell shape and integrity and is involved in cytoskeletal interactions such as adhesion and migration [36].

In this study we found GC cells lost their fibroblast-like appearance and acquired an epithelial morphology after treated with bufalin. Therefore, we also evaluated the expression of the EMT-relate genes E-cadherin and vimentin. Western blot analysis showed that bufalin increased E-cadherin expression and down-regulated vimentin expression; these results were more pronounced when cells were co-treated with bufalin and shRNA-ASCL2 (Figure 3C). Immunofluorescent detection also showed shRNA-ASCL2 and bufalin can increased E-cadherin expression and down-regulated vimentin expression (Figure 3D). Therefore, we speculated that bufalin suppresses EMT by down-regulating ASCL2 expression.

Wnt/β-catenin signaling pathways are crucial for cellular maintenance and development, include cell cycle progression, apoptosis, differentiation, migration and proliferation [37]. In addition, stimulation of these pathways is closely related to cancer development. During neoplastic transformation, the Wnt/β-catenin pathway is often aberrantly activated, increasing migration and invasion potential through increased β-catenin phosphorylation and/or nuclear localization [38]. Research suggested that Ascl2 is a master regulator of intestinal stem cell identity—deletion of the Ascl2 gene in the adult small intestine led to the disappearance of Lgr5 stem cells within day—and a Wnt target gene [16, 17, 39]. Therefore, we inhibited the Wnt signaling pathway with XAV939, which down-regulated β-catenin and ASCL2 and up-regulated E-cadherin; combining XAV939 with bufalin further decreased the expression of β-catenin and ASCL2 (Figure 4A). We also found that the migration ability of GC cells obviously decreased after XAV939 treatment, which increased the anti-invasion activity of bufalin (Figure 4B).

To explore the mechanism underlying the anti-GC activity of bufalin, we evaluated the effect of bufalin and ASCL2 on GC apoptosis *in vivo* and *in vitro*. *In vivo* study we found Bufalin decreased ASCL2 expression in the transplanted tumors and induced gastric cancer xenograft apoptosis. Then we detected the expression of apoptosis relate genes Bax, Bcl-2 and Caspase-3. We found after treated with Bufalin or down-regulate ASCL2 expression can increase Bax and Caspase-3 expression, at the same time down-regulated the expression of Bcl-2. And with more pronounced results upon combining bufalin and shRNA-ASCL2. Therefore, we inferred that bufalin inhibits GC tumorigenic behavior *in vivo* by influencing both cell proliferation and apoptosis, but the mechanism requires additional research.

Together, this study revealed that bufalin has anti-invasion and anti-metastasis activity in GC, and the underlying mechanism may relate to down-regulation of the Wnt/β-catenin signaling pathway, followed by the inhibition of ASCL2 expression and EMT. However, this mechanism requires additional elucidation and confirmation in future studies.

## MATERIALS AND METHODS

### Cell culture and materials

The human GC cell line AGS was obtained from the Shanghai Institutes for Biological Sciences and cultured in RPMI-1640 medium supplemented with 10% FBS (Gibco USA), 100 units/ml penicillin, 100 μg/ml streptomycin, and 2 mM glutamine at 37°C with 5% CO_2_. Bufalin (Sigma, USA) was dissolved in absolute ethyl alcohol and diluted in PBS. Oxaliplatin was obtained from Sanofi-Aventis (France); antibodies against E-cadherin, vimentin, MMP2, MMP9, and GAPDH were purchased from Cell Signaling Technology (Massachusetts, USA); and the ASCL2 antibody was obtained from Millipore (Massachusetts, USA).

### MTT assays

Cell proliferation was measured using the Cell Proliferation Reagent Kit I (MTT) (Roche, Basel, Switzerland). Logarithmic phase GC cells were seeded into 96-well plates (1×10^4^/well) in maintenance medium and then treated with different concentrations of bufalin (0, 50, 100, 200, 400, 1000, or 2000 nM) or oxaliplatin (0, 1.25, 2.5, 5, 10, or 20 μM); each concentration was evaluated in six wells. Cell proliferation was monitored at 0, 24, 48 and 72 h according to the manufacturer's instructions.

### Colony formation assays

The colony formation assay was performed as follows. Briefly, logarithmic phase GC cells were seeded into 6 cm dishes at approximately 2000 cells per dish, cultured at 37°C with 5% CO_2_ for 14 days, fixed in 4% formalin for 15 min, stained with Giemsa stain for 30 min, washed in water and dried. The colonies were observed by microscopy and qualified with ImageJ software. The colony formation rate was calculated using the following formula: colony formation rate (%)=colonies/plated cells×100%.

### Wound healing assays

Wound healing assays were conducted as previously reported [40]. A wound was created in a confluent layer of cells using a 200-μl pipette tip, and floating cells were removed by washing with PBS. The medium was replaced with fresh medium. Cells were subjected to the indicated treatment for 24, 48 or 72 h, and cells that migrated from the leading edge were photographed. Surplus cell migration area were analysed.

### Transwell assays

Cell migration and invasion were observed in transwell assays. Briefly, after treating cells with related drugs or interventions for 48 h, transwell assays were performed. First, the cells were adjusted to approximately 1×10^5^ cells/ml in serum-free medium, 150 μl of the cell suspension was added to the upper chamber, and 600 μl of DMEM containing 20% FBS was added to the lower chamber. The cells were incubated in the transwells for 48 h, and the transwell insert was then removed. A cotton-tipped applicator was used to carefully remove the cells that had not migrated/invaded from the top of the membrane. The membrane was washed twice with PBS, fixed with 3.7% formaldehyde for 15 min, and stained with crystal violet. The cells were observed and photographed under a light microscope.

### Stable shRNA-mediated knockdown of ASCL2 in cell lines

To knock down ASCL2, an shRNA sequence (5’-GCGTGAAGCTGGTGAACTTGG-3’) were designed and synthesized by GenePharma (Shanghai, China). A scramble shRNA was used as a negative control. The sequences were cloned into the lentiviral pLKO.1 puro vector, which was purchased from GenePharma (Shanghai, China). Lentiviral production and transduction were performed according to the manufacturer's protocol. Then, stable cells expressing lower ASCL2 levels were cultured, and western blot assays were used to verify the knockdown.

### Western blot

Total protein was extracted in RIPA buffer supplemented with protease and phosphatase inhibitors (Beyotime, JiangShu, China). Protein concentration was determined using the BCA protein concentration determination kit (Beyotime, JiangShu, China). Protein from the control and treated cell lysates was loaded onto 8-12% gradient NuPAGE gels (Novex, San Diego, CA, USA), electrophoresed under reducing conditions, and transferred onto polyvinylidene difluoride membranes (0.22 μm; Millipore, Billerica, USA). Western blot analysis was performed as described previously [25]. ASCL2 Monoclonal Antibody (MAB4417, Millipore, Billerica, USA), E-cadherin, vimentin, MMP2, MMP9, Bcl-2, Bax, Caspase-3 and GAPDH antibody (Cell Signaling Technology, MA, USA) were used.

### RNA isolation and quantitative RT-PCR

Total RNA was extracted using the TRIzol Reagent (Invitrogen, Carlsbad, CA, USA), and total RNA was reverse-transcribed with the PrimeScript RT-PCR Kit (TakaraBio, Otsu, Japan). The RT-PCR was performed using SYBR^®^ *Premix Ex Taq*™ II (Takara Bio, Otsu, Japan). The ABI 7500 real-time PCR system (Applied Biosystems, Irvine, CA, USA). The primer sequences used were as follows: forward 5′GCGTGAAGCTGGTGAACTTG-3′ and reverse 5′-GGATGTACTCCACGGCTGAG-3′ for *ASCL2*; forward 5′-GGAGCGAGATCCCTCCAAAAT′ and reverse 5′-GGCTGTTGTCATACTTCTCATGG-3′ for GAPDH. The 2^−ΔΔCT^ equation was used to calculate the relative expression levels.

### Immunofluorescence

Briefly, cells were fixed with 4% paraformaldehyde and permeabilized with 0.1% Triton X-100 in PBS. Following cell fixation, cells were incubated with the appropriate primary antibodies (vimentin and E-cadherin) in a solution of PBS with 1% bovine serum albumin and 0.1% Triton X-100 at 4°C overnight. Cells were further incubated with Alexa Fluor 488 labeled secondary antibody for 1 hour at room temperature, nuclei were stained with DAPI for 5 min. Fluorescent signals were detected by confocal fluorescence microscopy (LEICA DM IRB; Leica, Wetzlar, Germany)

### *In vivo* study

For the *in vivo* studies, 5- to 6-week-old male BALB/c mice (18–20 grams) were purchased from the Shanghai Laboratory Animal Center (SLACCAS, Shanghai, China). The mice were housed in a temperature- and humidity-controlled, pathogen-free animal facility and fed according to standard instructions provided by SLACCAS. All animal studies were performed in accordance with the International Standards of Animal Welfare and were approved by the Institute of Animal Care and Use Committee of Shanghai University of Traditional Chinese Medicine (approval number: SYXK, Shanghai, 2013-0055).

Gastric AGS cells, shRNA-control AGS cells, and shRNA-ASCL2 AGS cells were harvested from culture flasks and suspended in culture medium at a concentration of 1×10^7^ cells/ml. For each mouse, 0.2 ml of the cell suspension was injected subcutaneously into the right axillary region. After 3 weeks, the mice were randomly divided into the control group (AGS), shRNA-control group, shRNA-ASCL2 group, bufalin group, bufalin+shRNA-ASCL2 group, oxaliplatin group and oxaliplatin+shRNA-ASCL2 group, with at least five mice in each group. The intervention groups were treated with bufalin or oxaliplatin via intraperitoneal injection twice per week for 3 weeks. The mice were then sacrificed, and the tumors were excised. The tumor weight, maximum diameter (*a*) and minimum diameter (*b*) were measured, and the tumor volume (*V*) were calculated as follows: volume=*a*^2^*b*/2. Tumor tissue was stored in liquid nitrogen.

### Immunohistochemistry (IHC)

Immunohistochemical analysis was performed as described previously [25]. Briefly, tumor sections were stained with mouse anti-ASCL2 or mouse anti-MMP2 antibody at 4°C overnight. Goat anti-rabbit or anti-mouse IgG/horseradish peroxidase was applied as the secondary antibody according to the standard protocols provided by the manufacturer of the Elivision^TM^ plus kit (Maixin Bio, Fuzhou, China). For negative controls, the primary antibody was replaced with PBS. Five high power microscopic fields (×200) were randomly chosen from each slide to determine the positive staining intensity by IPP software (Image-Pro Plus 6.0, Media, Cybernetics). An unstained region was selected and set as the background. The protein expression level is presented as the average staining intensity of the 5 fields from each slide.

### Apoptosis assays

To determine apoptosis in tissue samples, TUNEL assays (terminal deoxynucleotidyl transferase deoxy-UTP-fluorescein nick end labeling assay) were performed. Tissues sections (4 *μ*m) were evaluated using the *In Situ* Cell Death Detection Kit (Roche, Mannheim, Germany) according to the procedures described by the manufacturer. Under a fluorescence microscope, apoptotic cells emit red fluorescence; 5 randomly chosen fields (×200) without any necrotic areas were observed, and the average percentage of positive cells in the 5 fields for each section was calculated.

### Statistical analysis

All data are presented as the percentage, mean and standard deviation (x ± sd), or median and 95% confidence interval (95% CI). Statistical analyses were performed using SPSS 17.0 for Windows. The two-tailed Student's t-test was used to analyze significant differences between groups. P<0.05 indicated statistical significance.

## Abbreviations

IHC: immunohistochemistry
GC: gastric cancer
NS: normal saline
EMT: epithelial-mesenchymal transition
ASCL2: Achaete-scute-like 2
MMP2: matrix metalloproteinase-2
MMP9: matrix metalloproteinase-9
CRC: colorectal cancer
ECM: Excetral Cellular Matrix
TUNEL: Terminal deoxynucleoitidyl Transferase Mediated Nick End Labeling
